# Physicians’ Mutual Benefit Association of Texas

**Published:** 1886-01

**Authors:** 


### SOCIETY NOTES.

### P. M. B. A. OF TEX.

At the date of the death of Dr. W. H. Park, holder of certificate No. 26, in this Association, there were sixty-one members in good standing. Assessments of one dollar each were issued to them under the rules, and, in accordance with their written pledges, the amount collected to constitute the benefit for the widow or other beneficiary. The following is a complete list of those who paid the assessment. It will be seen that four members—prominent, well-known physicians—failed to do so, and thus forfeited their membership; their names are omitted from this list. Since the above date some forty-five new members, or seventy per cent. of the whole numbers, at this writing, have joined, and the Association has received a new impetus, and now bids fair to become useful:

Dr. W. P. Burts, Ft. Worth.
A. S. Wolff, Brownsville.
H. C. Ghent, Belton.
S. W. Sholars, Orange.
Geo. Cupples, San Antonio.
F. Herff, "
D. V. Spring, Khrone P. O.
P. Jourdan, Beaumont.
R. M. Swearingen, Austin.
H. W. Brown, Waco.
R. G. Williams, Whitney.
M. M. Myers, Khrone.
C. E. R. King, San Antonio.
B. G. Hadra, Austin.
W. M. Powell, Albany.
J. W. McLaughlin, Austin.
M. A. Taylor, "
F. E. Daniel, "
W. A. Morris, "
T. J. Bennett, "
C. O. Weller, "
J. A. Throckmorton, Houston.
A. R. Kilpatrick, Navasota.
T. J. McFarland, Indianola.
J. W. Daniel, Houston.
F. A. Tompkins, Sandy Point.
J. T. Webb, Terrell.
Y. D. Harrington, Terrell.

Dr. R. H. Day, Baton Rouge.
J. W. Dupre, " "
S. A. Towsey, Galveston.
J. P. Coffey, Plano.
W. F. Buck, Pecos.
S. F. Starley, Tyler.
T. M. Matthews, Athens.
M. D. Sterrett, Grand Bluff.
F. T. Paine, Comanche.
G. W. Gray, Terrell.
A. H. S. Hardin, "
J. M. Ball, New Boston.
R. W. Reid, "
W. T. Strain, Elmo.
E. S. Tidwell, "
J. M. Ross, Brenham.
A. M. Hill, Hill's Prairie.
C. C. Francis, Cleburne.
H. H. Thorp, Liberty Hall.
J. M. Hons, Burton.
J. R. Scales, Henderson.
L. H. Hardy, Paige.
A. D. Stroud, Henderson.
L. J. Graham, "
John Inabnit, Terrell.
A. D. Paulus, Shulenberg.
M. B. Pollard, Terrell.
Total, 55.

Since writing, Dr. J. B. Duchine and Dr. W. H. Pyle have each paid $1.

Below find correspondence with Mrs. Park, and her receipt:

PHYSICIANS' MUTUAL BENEFIT ASSOCIATION OF TEXAS,
OFFICE OF SECRETARY AND TREASURER,
AUSTIN, TEXAS, Dec. 24, 1885.

MRS. W. H. PARK, Tyler, Texas:

*Dear Madam:—*

It becomes my pleasant duty, as Secretary of the Physicians' Mutual Benefit Association of Texas, of which Dr. Park was an honored member, and holder of certificate No. 26, to hand you, herewith enclosed, a sight draft on Galveston for $55, this being the total amount collected by assessment of $1 on each surviving member in good standing at the time of Dr. Park's death, November 4, 1885, under the Constitution and By-laws of the Association.

This amount I beg you to accept as a free-will offering from a small brotherhood of physicians, representing, as far as the number goes, the better element of a profession of which your lamented husband was an acknowledged ornament—accompanied by the assurance of sympathy on the part of the members of the Association in your bereavement.

Enclosed I also send you a receipt, which please oblige me by signing, and have Dr. Starley return it.

Very respectfully, your obedient servant,

F. E. Daniel, M. D.,

Secretary and Treasurer, P. M. B. A., of Texas.

Tyler, Texas, Dec. 29, 1885.

Dr. F. E. Daniel, Austin, Texas:

*Dear Sir:—*

Yours with draft enclosed received. Enclosed please find receipt. Please return my heartfelt thanks to the members of the Mutual Benefit Association for their kindness, also my kind regards to you, doctor, for your trouble.

Truly your friend,

Mrs. W. H. Park.

$55.00

Received, Tyler, Texas, Dec. 25, 1885, of Dr. F. E. Daniel, Secretary and Treasurer of the Physicians' Mutual Benefit Association of Texas, fifty-five dollars, the total amount paid in under assessment No. 1, for the benefit of certificate No. 26, for the benefit of my late husband, Dr. W. H. Park.

Mrs. W. H. Park.

